# Evaluation of antibacterial property and biocompatibility of Cu doped TiO_2_ coated implant prepared by micro-arc oxidation

**DOI:** 10.3389/fbioe.2022.941109

**Published:** 2022-09-01

**Authors:** Binbin kang, Dongmei Lan, Chao Yao, Ping Liu, Xiaohong Chen, Shengcai Qi

**Affiliations:** ^1^ School of Materials and Chemistry, University of Shanghai for Science and Technology, Shanghai, China; ^2^ Medical College, Anhui University of Science and Technology, Huainan, China; ^3^ Department of Prosthodontics, Shanghai Stomatological Hospital, Fudan University, Shanghai, China; ^4^ Shanghai Key Laboratory of Craniomaxill of Acial Development and Diseases, Fudan University, Shanghai, China

**Keywords:** Cu doped, osteogenic differentiation, antibacterials, micro-arc oxidation, TiO_2_ coating

## Abstract

In order to enhance osteogenic differentiation and antibacterial property of dental implants, volcano-shaped microporous TiO_2_ coatings doped with Cu were fabricated via micro-arc oxidation (MAO) on Ti. Cu-doped coating with different mass ratios of Cu were obtained by changing the concentration of copper acetate in the electrolyte. The structure of Cu-TiO_2_ coatings were systematically investigated. Element Copper was uniformly distributed throughout the coating. Compared with TiO_2_ coating, the Cu-doped can further improved proliferation of bone mesenchymal stem cells (BMSCs), facilitated osteogenic differentiation. The bacteriostasis experiments demonstrated that Cu-doped TiO_2_ coating possess excellent antibacterial property against *Staphylococcus aureus* (*S. aureus*) and Porphyromonas gingivalis (*P. gingivalis*).

## 1 Introduction

Titanium and titanium alloys are widely used for orthopedic implants including joint prostheses, fracture fixation devices and dental implants, due to their excellent mechanical properties and biocompatibility (Elias et al., 2008). In dental implants, Ti implants are used for missing teeth replacement more frequently (Qi et al., 2019). Dental implants have shown high survival rates of up to 99% over 10 years. Despite the high success rates of dental implants, the major reason for implant failure is peri-implantitis, which affect both the surrounding hard and soft tissue. Due to prevalence rates up to 56%, peri-implantitis can lead to the loss of the implant without multilateral prevention and therapy concepts (Smeets et al., 2014). The treatment of peri-implantitis infections comprises conservative (nonsurgical) and surgical approaches, while the prognosis of peri-implantitis therapy is far away from satisfactory today (Heitz-Mayfield et al., 2014). Especially for serious cases, the only way is to remove contaminated implants from the patient (McConoughey et al., 2014; Tsutsumi et al., 2021). The primary etiologic reason for the inflammation of peri-implant tissues is the oral biofilm (Sanz et al., 2012). Once bacteria adhere to the surfaces of dental implants, a biofilm which makes the bacteria highly resistant will be formed (Zhu et al., 2013). The formation of the biofilm usually includes the initial fast stage attached by the microbe on the surface, multi-layered bacterial proliferation, intracellular adhesion in an extracellular polysaccharide matrix and the intercellular adhesion stage (Trampuz et al., 2003). Therefore, inhibiting bacterial adhesion and initial proliferation are essential to prevent infection around implants (Thukkaram et al., 2020).

The surround tissue integration and antibacterial property at the hosting tissue-implant interface are important to the performance life of an implant (Brunello et al., 2019). The remarkable bio-compatibility and convenient osseointegration is needed for the implant. The coating on dental implant will modify the surface topography and chemistry to improve osseointegration and thereby increase treatment predictability (Milleret et al., 2019). The appropriate surface topography affects the biological viability of tissue and bone cells to promote osseointegration (Elter et al., 2008). There are many methods to modify the surface of dental implant, such as loading with biocompatible coating (Wei et al., 2022), like polydopamine (Zeng et al., 2020), sintering (Manière et al., 2020), HA coating (Kim et al., 2021; Montoya et al., 2021), Titanium Plasma Spray (Wang et al., 2020), Sandblast Large grit and Acid-etching (SLA) (Kim et al., 2017), anodic oxidation (Wu et al., 2019) and micro-arc oxidation (MAO). Among these techniques, MAO maybe one of the most cost-effective and versatile ways to modify the surface of metallic implants with complex geometries (Durdu, 2018). MAO results in a changed surface topography and an increased thickness of the native oxide layer, which is suitable for osseointegration. The most striking character of MAO is the formation of interconnecting pores, which can increase protein adsorption and stimulate osteoblast migration resulting in faster osseointegration (Hu et al., 2010a). Therefore, MAO maybe a potential technique to modify the dental implant.

In addition to good bio-compatibility, antibacterial properties also needed for the implant. The strategy to prevent biofilm formation is the application of antibacterial coating on the dental implant for prevent the peri-implantitis. Addition of antimicrobial coating of biomaterial surfaces is highly desirable to offer antibacterial activities and prevent implant-associated infections (Zhu et al., 2013). Generally, inorganic antibacterial metallic like Ag, Au, Cu, Zn, and Mn, are among the most widely used antimicrobial agents (Mei et al., 2014; Zhang et al., 2018a; Alqattan et al., 2021; Gui et al., 2021). Among these elements, Ag cannot be used due to the potential issues of biosafe, while Cu, an essential trace element for human body generally has limited *in vivo* cytotoxicity, which also possesses an increased antibacterial efficacy *in vivo* (Zhang et al., 2021). Besides, Cu is one of essential elements in living organisms and also play diverse roles in human health (Jia et al., 2012). Hence, Cu was used in original electrolyte to form the Cu-doped coating improve the antibacterial capability of implants by MAO in our study.

In this paper, the crateriform nanostructured Cu doped titanium dioxide coatings (Cu-MAO) were prepared on Ti by the MAO process. The characterization of Cu-MAO was evaluated by SEM, EDS, XRD, XPS, CLSM, and ICP-OES. The proliferative and osteoblastic capacities of BMSCs were investigated *in vitro*. The antibacterial capacity of the coatings against *P. gingivalis* and *S. aureus* were also evaluated *in vitro*.

## 2 Experimental

### 2.1 Materials and preparation of Cu-doped TiO_2_ coating (Cu-MAO)

Titanium samples were cut from commercial pure titanium (cp-Ti, TA4) rod and gradually polished by 1200 # SiC sandpaper. Then the samples were ultrasonically washed with acetone, absolute ethanol and distilled water prior to treatment.

An aqueous electrolyte consisting of 20 g/L trisodium phosphate dodecahydrate (Na_3_PO_4_•12H_2_O), 4 g/L sodium hydroxide (NaOH), 5 ml/L glycerol and 10 g/L EDTA disodium was prepared as the original electrolyte (Group MAO), while the experimental electrolytes (Group 0.5Cu-MAO, Group 1Cu-MAO, and Group 2Cu-MAO) were prepared by adding 0.5 g/L, 1 g/L, and 2 g/L of copper acetate [Cu(CH_3_COO)_2_·H_2_O] into the original electrolyte.

MAO was carried out by a DC power supply. A stainless-steel plate was used as the cathode plate and the titanium sample as the anode. The temperature of electrolyte was maintained at 30°C via circulating water treatment system. The fabrication was conducted under the constant voltage of 400 V for 5 min. After that, the samples were ultrasonically washed with deionized water for 5 min, dried and stored at room temperature until use.

### 2.2 Characterization of Cu-MAO

The surface and cross-sectional morphologies of coatings were observed by a scanning electron microscope (SEM, Quanta FEG 450). The element compositions of the coatings were analyzed by an energy dispersive X-ray spectroscopy (EDS). X-ray diffraction (XRD, D8 ADVANCE, Bruker, Germany) was conducted to analyze phase composition. Using X-ray microanalysis system (XPS) analyzed superficial composition of the coatings. The hydrophilicity of Cu-MAO was analyzed using a contact angle meter. Three samples from each group were measured to obtain the average. Besides, the surface roughness of those coatings was measured using confocal laser scanning microscope (CLSM). Those Cu-doped samples were immersed in 10 ml physiological saline solution at 37°C for 28 days, and the concentrations of Cu^2+^ ions in each coating immersed solution were measured via inductively coupled plasma-optical emission spectrometry (ICP-OES).

### 2.3 *In vitro* cell compatibility assay

BMSCs were isolated and cultured from the bone marrow of 4-week-old Sprague–Dawley (SD) rats. Briefly, bone marrow was isolated from tibias of mice and then suspended in alpha minimum essential medium (α-MEM, Gibco, United States) containing 10% fetal bovine serum (FBS, Gibco, United States) and 1% penicillin-streptomycin (PS, Gibco, United States). Primary cells were cultured for 48 h at 37°C in a humidified atmosphere of 5% CO_2_. Then BMSCs adhered to the bottom of the dishes were collected. The medium was replaced every 3 days. Cells between passage 3 and 6 were used for the following experiments.

Osteogenic differentiation was induced by osteogenic medium, which contains 10% FBS, 1% penicillin/streptomycin, 50 μg/ml ascorbic acid (Sigma, United States), 10 mM sodium β-glycerophosphate (Sigma, United States) and 10 nM dexamethasone (Sigma, United States). The cells were maintained by the addition of fresh osteogenic induction medium every 3 days.

#### 2.3.1 Cell viability, adhesion and morphology assays

For cell viability assays, all the sterilized Cu-MAO samples (Diameter = 14 mm) were placed individually into the 24 well plates. After samples were rinsed twice with PBS, cells were seeded onto the samples at a density of 7×10^4^ cells/ml. Cytotoxicity was measured after 1, 3, and 5 days of culture. The medium was changed every other day during proliferation trials. After the prescribed incubation time, CCK-8 dye was added to each well, then the plates were cultured for another 2 h at 37°C. At last, the optical density (OD, *n* = 5) was measured at 450 nm by a plate reader. Five samples were employed for each coating for the test. The cell viability was calculated using the following equation:

$$Cell\;Viability\;\left(\%\right)=\;\frac{OD_{sample}-OD_{blank}}{OD_{control}-OD_{blank}}$$ (1)

The cells after incubated for 3 days were washed with PBS, fixed with 4% Paraformaldehyde for 10 min at room temperature and then dehydrated in ethanol for 10 min per gradient, followed by vacuum drying. After gold sputtering, the samples were observed via SEM (Quanta FEG 450) for cell growth morphologies.

#### 2.3.2 Osteogenic differentiation assays of antibacterial coatings

The sterilized Cu-MAO samples (Diameter = 30 mm) were placed individually into the 6-well plates. 2ml of the BMSCs cell suspension was plated into the 6-well plates at a density of 1 × 10^5^ cells/ml overnight. Then each well was replaced with the osteogenic medium for 0 and 7 days. After 7 days induction, Total RNA was isolated with the Trizol (Invitrogen, United States) extraction method according to the manufacturer’s protocol and measured by a Nanodrop system (Thermo Fisher Scientific, United States). CDNA was reverse transcribed with a PrimeScript^TM^ RT Master Mix (TaKaRa, Japan). RT-PCR was conducted with Hieff^®^qPCR SYBR Green Master Mix (Yeasen, Shanghai). Relative gene expression was calculated using the comparative 2^–ΔΔCT^method. The primers sequences are listed in Table 1.

**TABLE 1:** Nucleotide sequences of primers used for RT -PCR.

Gene	Forward primer (5′-3′)	Reverse primer (5′-3′)
*Alp*	AAC​GTG​GCC​AAG​AAC​ATC​ATC​A	TGT​CCA​TCT​CCA​GCC​GTG​TC
*Opn*	CCATTTACGGAGACCCAC	TCTGAGCGGCAACTTTAT
*Ocn*	TGA​GGA​CCC​TCT​CTC​TGC​TC	GGG​CTC​CAA​GTC​CAT​TGT​T
*Runx2*	GCA​CCC​AGC​CCA​TAA​TAG​A	TTGGAGCAAGGAGAACCC

Gene abbreviation: *Alkaline phosphatase (Alp), Osteopontin (Opn)*, *Osteocalcin (Ocn)*, *Runtrelated transcription factor 2 (Runx2).*

Meanwhile, total protein was isolated after culturing with the osteogenic medium for 0 and 7 days with RIPA lysis buffer (biosharp, Shanghai) containing protease inhibitor (biosharp, Shanghai) and phosphatase inhibitor (Beyotime, Shanghai). The protein concentration was measured using a BCA assay kit (Beyotime, Shanghai). GAPDH (Abcam, United States), ALP (Genxspan, Shanghai) and RUNX2 (CST, United States) were diluted at 1:5000, 1:500, 1:500 respectively in the nonfat powdered milk TBST solution and incubated with the membrane overnight in a 4°C chamber. After washing by 0.5% TBST, the membrane was incubated with rabbit anti-mouse IgG conjugated to HRP (Abcam, United States) diluted 1:2000. The bound antibody was detected using enhanced chemiluminescence. Three samples for each group were tested, and each test was repeated Three times. (*n* = 3)

Alkaline phosphatase (ALP) staining was performed using an ALP staining kit (Beyotime, China). After 7 days of induction, the cells were washed with PBS for 3 times. Then fixed with 4% Paraformaldehyde for 10 min and stained with ALP reagent for 10 min. After washed with PBS, the stained cells were subsequently observed. For Alizarin Red Staining, cells were cultured with the osteogenic differentiation medium for 14 days, and mineralization level in cells were determined by Alizarin red staining kits (Solarbio, Shanghai). After fixation, the cells were stained with 1% solution of Alizarin red for 10 min and washed with distilled water for three times. The stained cells were subsequently observed, with representative images captured.

### 2.4 *In vitro* antibacterial assay

The Gram-negative *P. gingivalis* (ATCC 33277) and Gram-positive *S. aureus* (ATCC 25923) were used to assess the antibacterial properties of the coatings. After cultured in brian heart infusion (BHI) at 37°C for 12 h. *P. gingivalis* and *S. aureus* were adjusted to a concentration of 10^6^ CFU ml^−1^. The diluted cell suspension (0.2 ml) was inoculated onto the surface of Cu-MAO. A sterilized sealing film was covered tightly on the surface of the sample to insure the anaerobic environment. Each sample was incubated in anaerobic incubator for 24 h. The micro-organisms were removed from the samples by 1 ml of PBS in a cell culture dish. Then, the above surplus diluted bacterial suspensions were spread on a columbia agar plate and were further incubated for 24 h anaerobic (*P. gingivalis*) or aerobic (*S. aureus*) culture. Finally, the colony-forming units (CFU) on the agar plate was counted for each group, and the antibacterial rate was calculated by the following formula (Su et al., 2022):

$$Antibacterial\;rate=\left(1-\frac{CFU_{sample}}{CFU_{control}}\right)\times 100\%$$ (2)

Where CFU_sample_ stand for the number of bacterial colonies on the 0.5Cu-MAO, 1Cu-MAO and 2Cu-MAO coating and CFU_control_ stand for the number of bacterial colonies on the 0Cu-MAO coating. The experiment was carried out three times, and CFU was the average of three results.

The Live/Dead staining was used to assess the antibacterial capacity of Cu-MAO against *S. aureus* biofilm. After incubation for 24 h, the samples were washed by PBS and stained by a Live & Dead Bacterial Staining Kit according to the manufacturer’s protocol with three replicates. The results were examined and photoed under the laser scanning confocal microscope (LSCM).

The bacterial growth morphology on Cu-MAO was observed by SEM. The bacterial cells after incubated on the surface for 24 h were washed with PBS, fixed with 4% Paraformaldehyde for 10 min at room temperature and then dehydrated in ethanol for 10 min per gradient, followed by vacuum drying. After gold sputtering, the samples were observed via SEM (Quanta FEG 450) for bacterial growth morphologies.

### 2.5 Statistical analysis

All the assays were conducted in triplicate and repeated at least three times. Data were collected and the significant differences were assessed using ANOVA followed by one-tailed Student’s t-tests. Statistical significance was considered at *p* < 0.05.

## 3 Results

### 3.1 Structure of Cu-MAO

**FIGURE 1:**
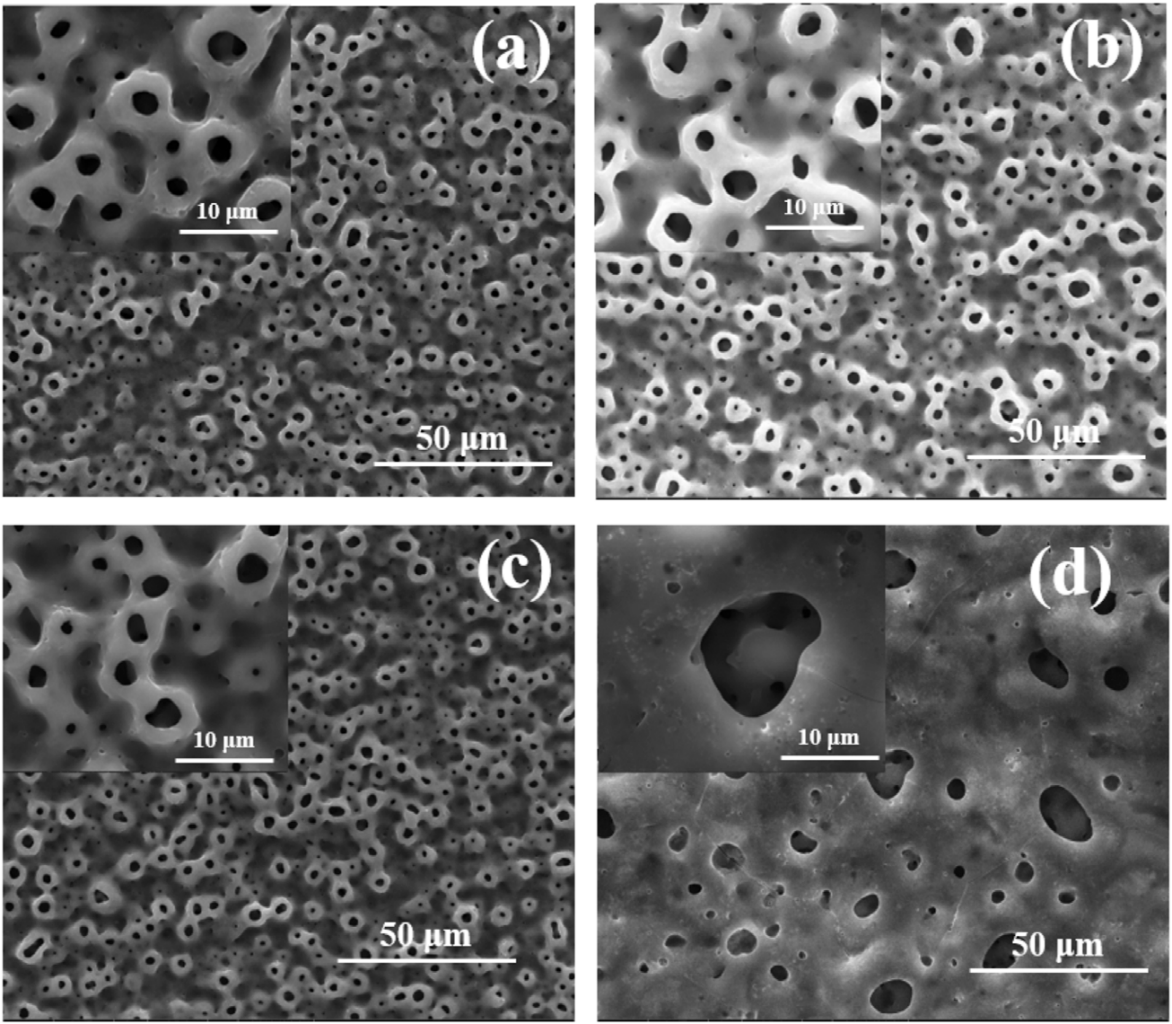
Surface morphologies of different coatings: **(A)** 0Cu-MAO, **(B)** 0.5Cu-MAO, **(C)** 1Cu-MAO, **(D)** 2Cu-MAO.

Figure 1 shows the surface morphologies of TiO_2_ coatings doped with different amounts of Cu under SEM, which indicated that all the Cu-MAO have a microporous structure. There was no significant difference in the surface morphology among 0Cu-MAO, 0.5Cu-MAO and 1Cu-MAO coatings (Figures 1A–C). Whereas, after the addition of 2 g/L copper acetate into the electrolyte, the TiO_2_ coating exhibited larger pore size and lower porosity in 2Cu-MAO (Figure 1D).

It can be seen from Table.2, EDS surface scanning analysis that the coating surfaces all contain Ti, O and P. As the increased concentrations of copper acetate added in the electrolyte, the amount of Cu increases from 0.47 (0.5Cu-MAO) to 2.87 Wt% (2Cu-MAO).

**TABLE 2:** Element component (wt%) on the surface of different coatings.

Sample	Element component (wt%)			
	Ti	Cu	O	P
0Cu	72.68	-	17.23	10.09
0.5Cu	71.44	0.47	16.20	11.90
1Cu	70.84	1.04	15.54	12.58
2Cu	63.73	2.87	17.54	15.86

**FIGURE 2:**
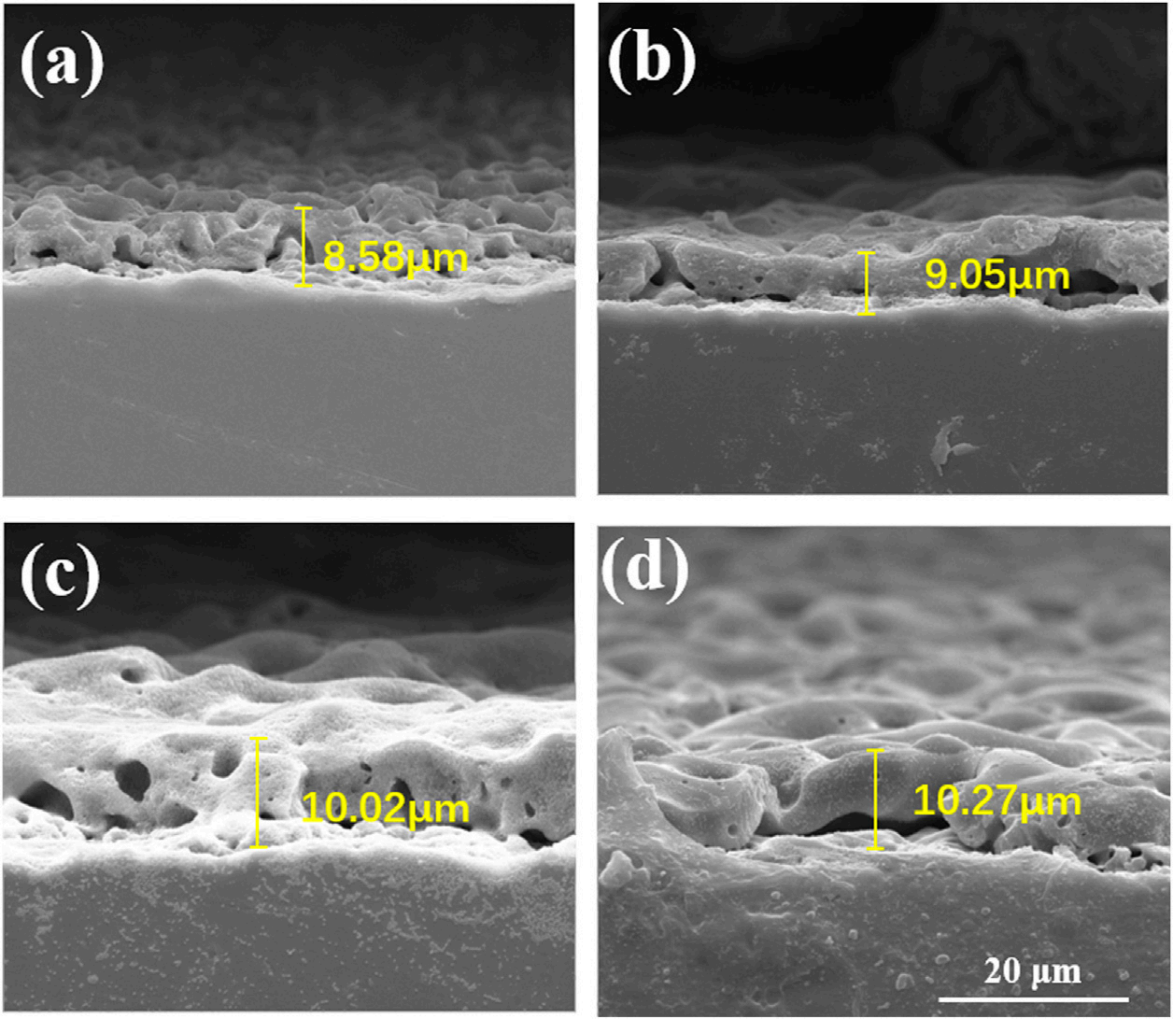
Cross-section morphologies of different coatings: **(A)** 0Cu-MAO, **(B)** 0.5Cu-MAO, **(C)** 1Cu-MAO, **(D)** 2Cu-MAO.

**FIGURE 3:**
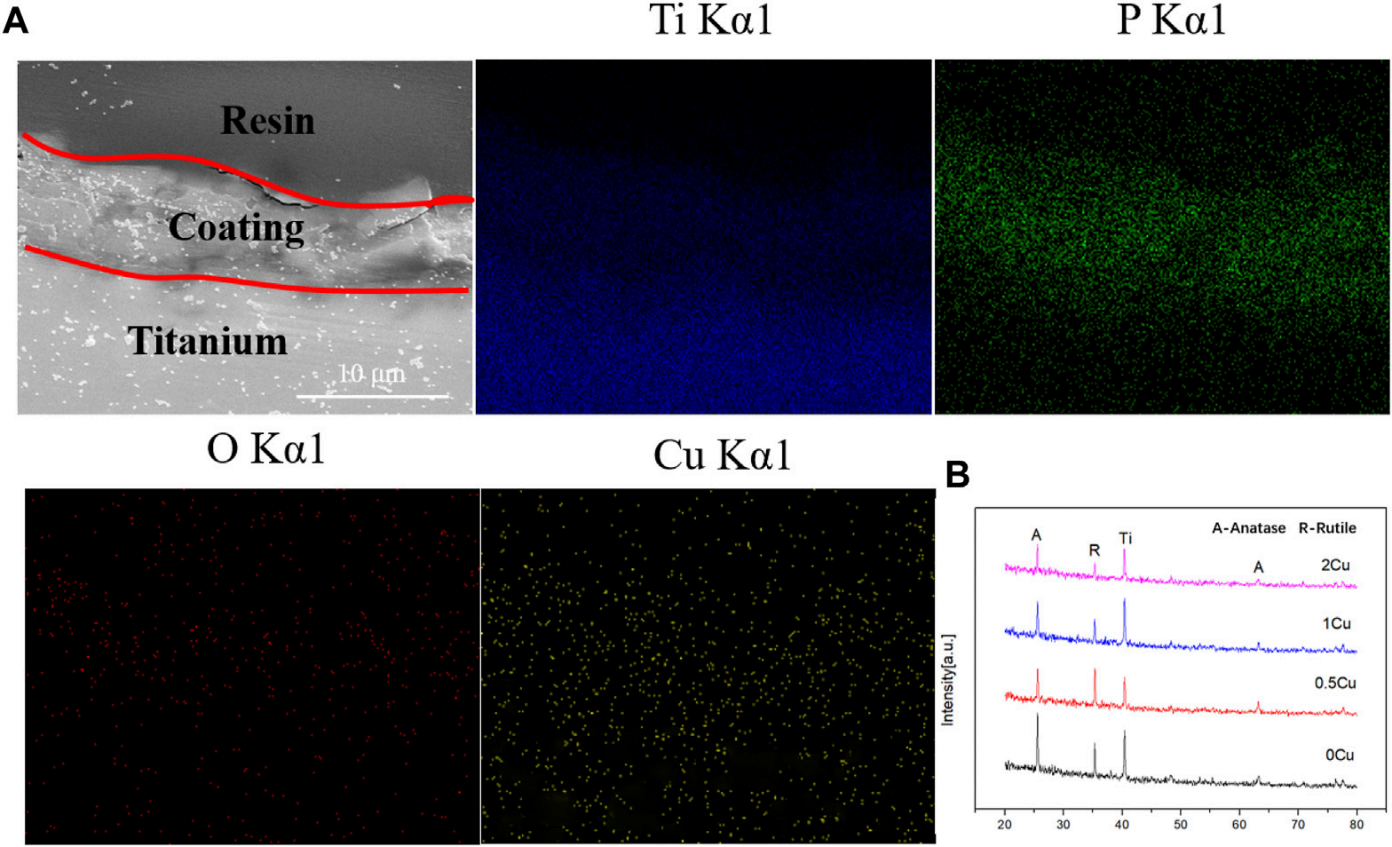
Cross-sectional backscattered electron morphology and elemental distributions of the 1Cu-MAO coating **(A)**. XRD patterns of 0Cu-MAO, 0.5Cu-MAO, 1Cu-MAO and 2Cu-MAOcoatings **(B)**.

The cross-section morphologies were shown in Figure 2. Both TiO_2_ coating and Cu-MAO coatings have a cross-sectional thickness of about 9∼10 μm, and the crater-like porous structure is clearly visible. There is no crack between the coatings and Ti substrates, indicating high adhesion of TiO_2_ and Cu-doped coatings. The EDS analysis of cross-sectional showed that Cu, Ti, O and P were uniformly distributed throughout the film in 2Cu sample (Figure 3A). The results of XRD showed that 0Cu-MAO, 0.5Cu-MAO, 1Cu-MAO, and 2Cu-MAO coatings mainly consisted of anatase and rutile (Figure 3B), and none of Cu contained compound is detected by XRD. The doping of copper did not alter phase compositions of the coatings.

**FIGURE 4:**
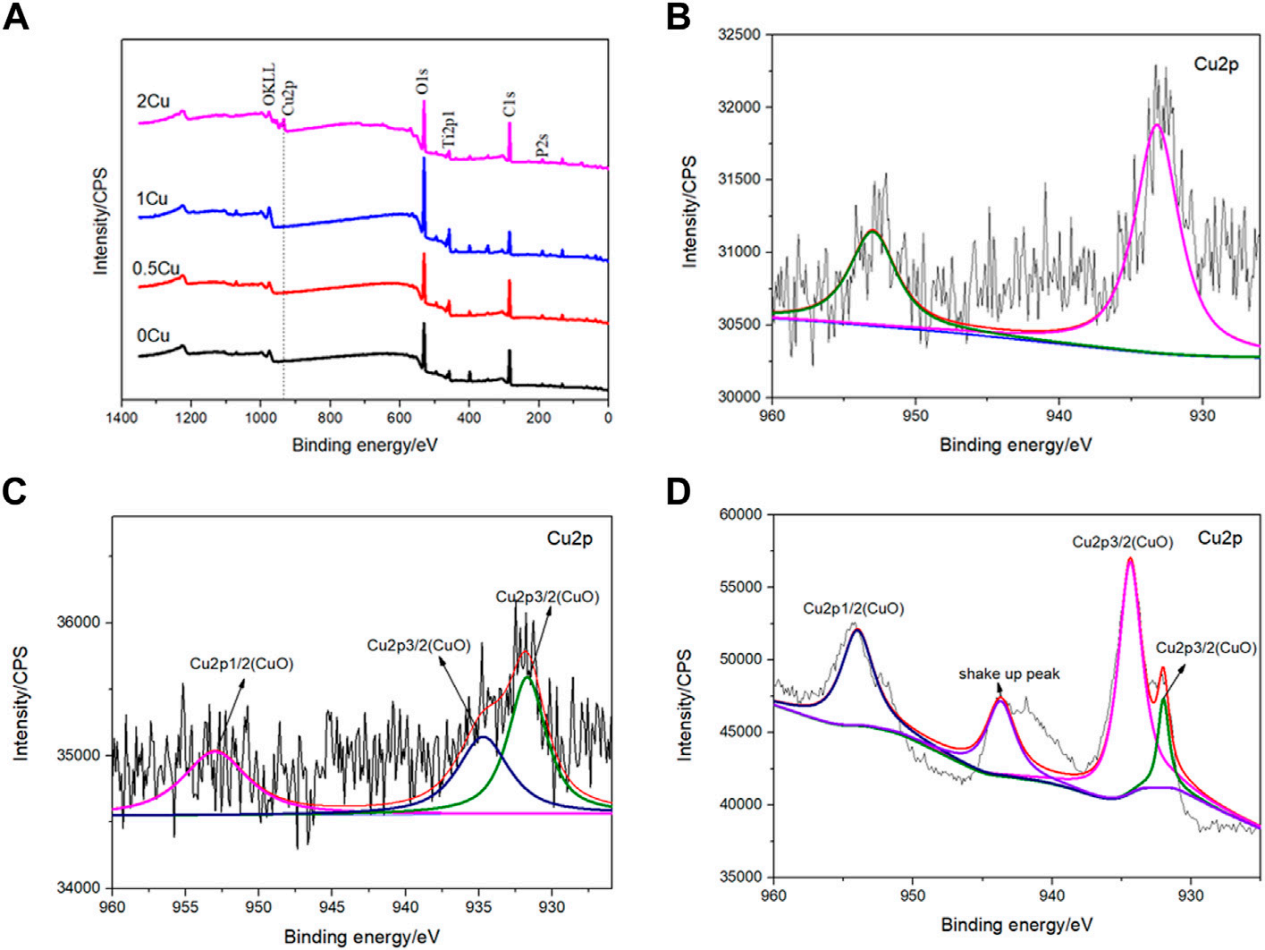
XPS survey spectrum **(A)** and Cu2p high-resolution spectra of the 0.5Cu-MAO **(B)**,1Cu-MAO **(C)**, 2Cu-MAO **(D)** coating.

For the purpose of analyzing the chemical statements of Cu in those coatings, XPS analysis was carried out. Figure 4A is the full spectra of different coatings, showing that the elements of 0.5Cu-MAO, 1Cu-MAO and 2Cu-MAO coating surface were Ti, O, P, and Cu. According to the full spectrum XPS results, we found that different copper acetate comcentrations did not change the XPS spectrum peaks in the four group of coatings. Beside the strength of Cu peak increases with the increased content of Cu in the coating. To further identify the chemical configurations of Cu, XPS high-resolution spectra are shown in Figures 4B–D. As for 0.5Cu-MAO coating, Cu 2p_1/2_ and Cu 2p_3/2_ appeared at 952.48 and 932.78 eV, respectively. There are three peaks of 1Cu-MAO at 953.08, 934.88 and 931.68 eV in Cu 2p high-resolution XPS spectrum. There were no shake-up peaks in the XPS spectrum of 0.5Cu-MAO and 1Cu-MAO coatings, indicated that Cu exsited primarily in Cu_2_O or metallic Cu (Rokosz et al., 2016). As indicated in Figure 4D, the binding energies of Cu 2p peaks at 955.9 ± 0.2 and 934.9 ± 0.2 eV and other satellite peaks suggest that Cu bond with O and exist as CuO in 2Cu-MAO coating (Wang et al., 2015).

**FIGURE 5:**
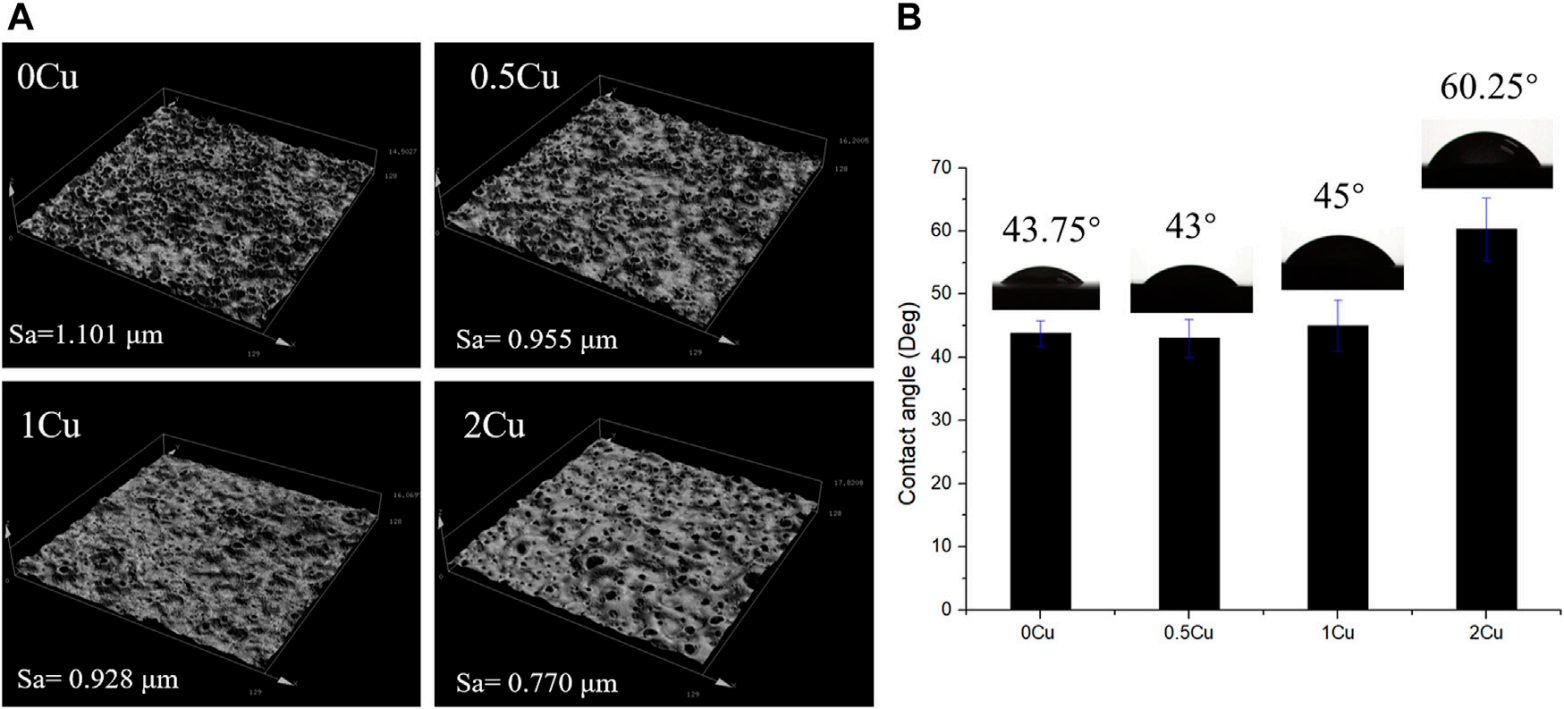
Confocal Laser Scanning Microscope images **(A)** and digital photograph of water droplets of different coatings **(B)**.

The results of CLSM revealed that there was no significant difference among 0Cu, 0.5Cu and 1Cu groups in the surface micro-roughness, and the value of Ra are all about 1 μm as shown in Figure 5 A. However, the addition of 2 g/L copper acetate into the electrolyte reduced the surface micro-roughness of the coatings. In additon, the 3D images intuitively revealed that a volcano structure appears on the surface of the coatings. The wettability of the coating was determined by water contact angle shown in Figure 5B. The contact angle of 0Cu-MAO, 0.5Cu-MAO and 1Cu-MAO coatings were in the range of 43–45°. Compared with 0Cu-MAO group, adding Cu do not obviously alter their hydrophilicity. But the contact angle of 2Cu-MAO coating was rised to 60.25°.

**FIGURE 6:**
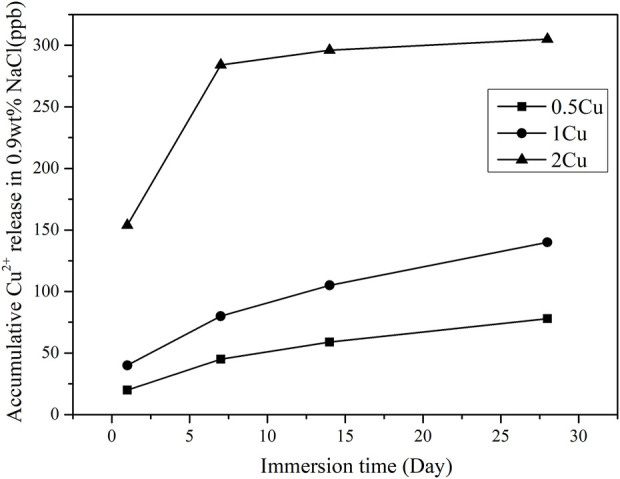
Cu^2+^ release kinetics from different coatings after immersed in 0.9 wt% NaCl for 1–28 days.

The coatings (0.5Cu-MAO, 1Cu-MAO and 2Cu-MAO) were immersed in 0.9 Wt% NaCl for 1–28 days, and the cumulative amount of Cu^2+^ released from different coatings were evaluated and shown in Figure 6. With the prolonged immersion process, Cu ions released fast at initial and then continuously in a low level. At the same immersion time, the cumulative concentrations of Cu^2+^ increased with the added amount of Cu in TiO_2_ coating.

### 3.2 The cell biocompatibility and osteogenic ability of Cu-MAO

**FIGURE 7:**
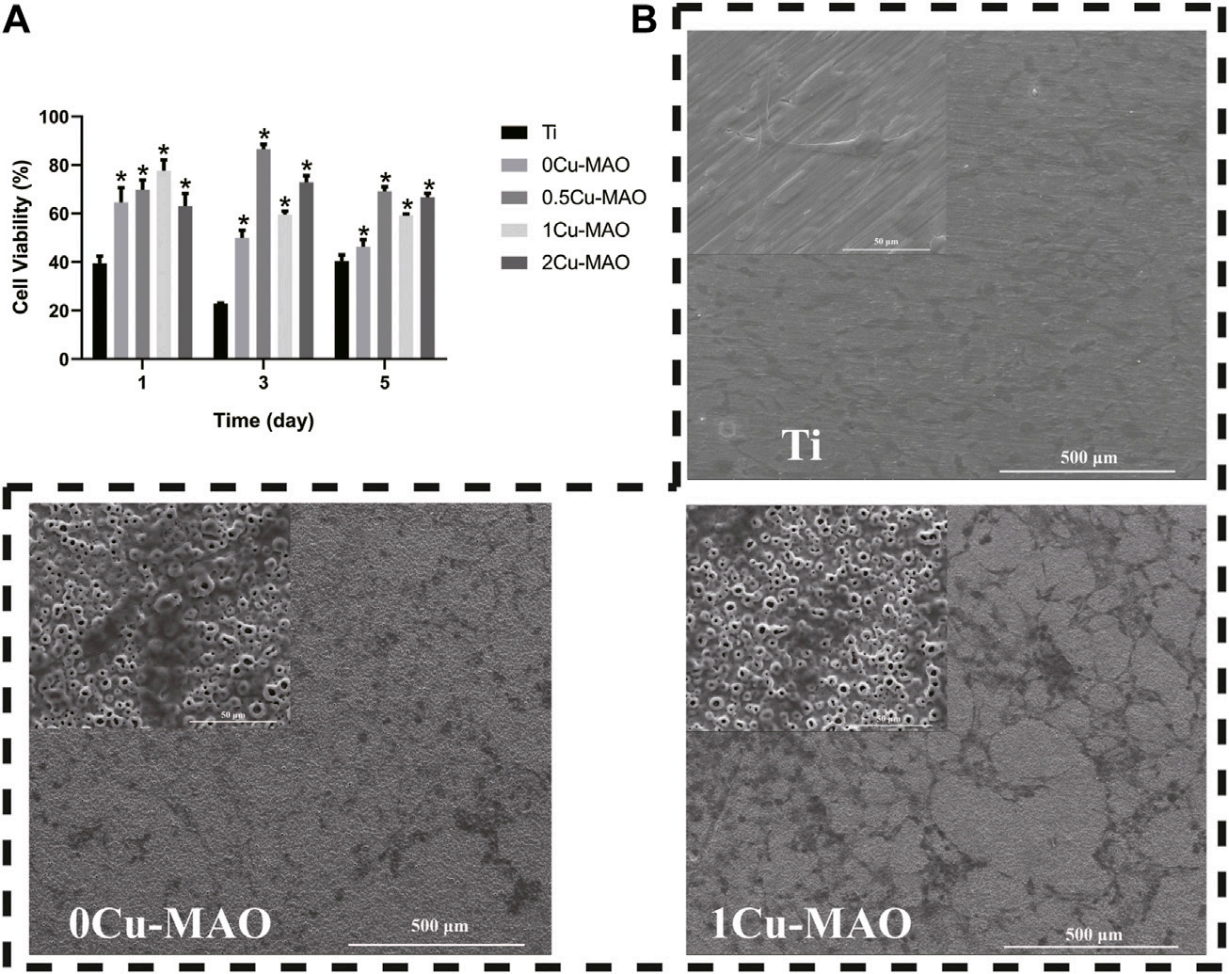
The cell biocompatibility of Cu-MAO. **(A)** Cytotoxicity was measured after 1, 3, and 5 days of culture. **(B)** SEM images of cell morphologies culture on different surface for 3 days. Data are presented as mean ± SD; (*p < 0.05, vs. Ti group).

The cell viablity was test by CCK-8, the result of which showed the OD value was increased significantly in 0Cu-MAO, 0.5Cu-MAO, 1Cu-MAO and 2Cu-MAO groups at 1, 3, 5 days (*p* < 0.05) (Figure 7A). Compared with 0Cu-MAO group, the 0.5Cu-MAO, 1Cu-MAO and 2Cu-MAO groups showed much higher viability at 3 and 5 days. All the resutls present the good cell biocompatibility of Cu-MAO coatings in our study. As shown in Figure 7B, the cell morphologies on different coatings after incubation for 3 days were observed by SEM. Cells were spread better on the surface of 0Cu-MAO and 1Cu-MAO. However, compared with 0Cu-MAO, more cells adhered on the 1Cu-MAO.

**FIGURE 8:**
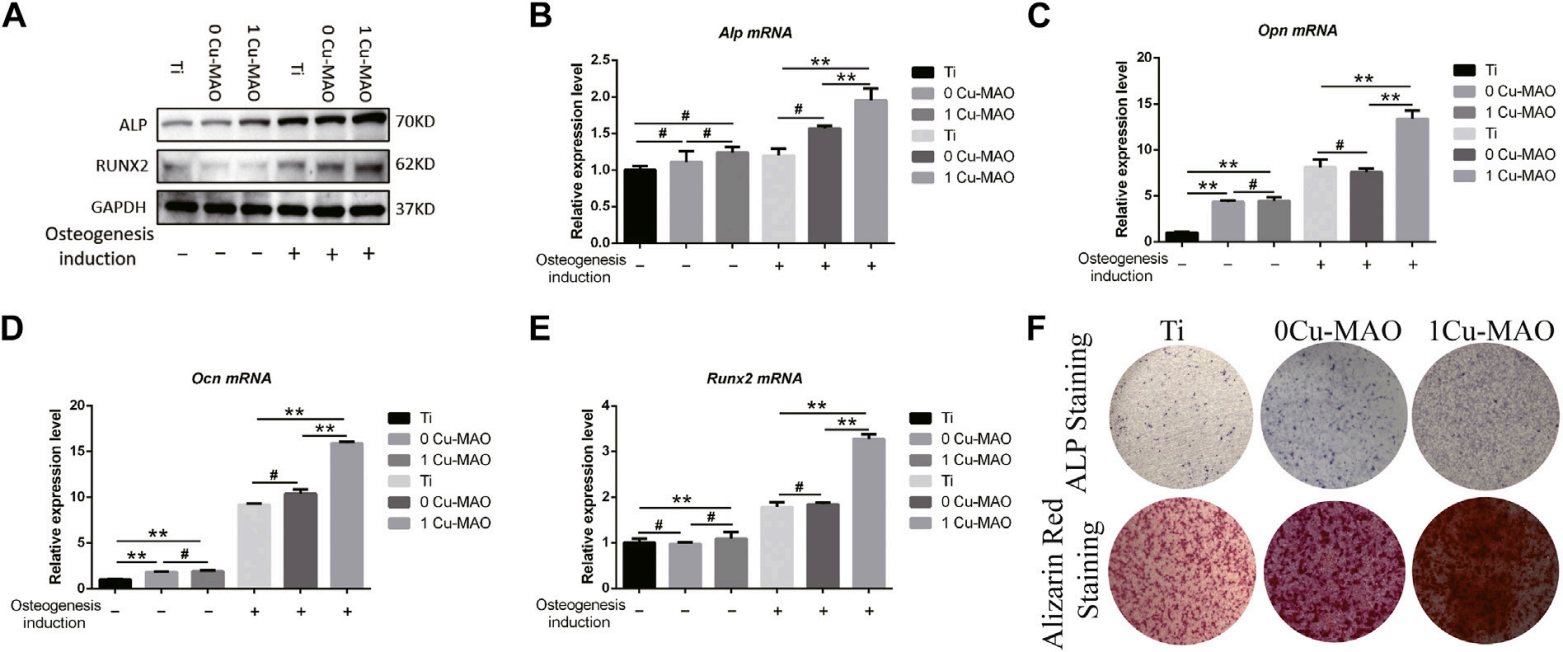
The osteogenic ability of Cu-MAO. **(A)** Osteogenic related protein (ALP and RUNX2) expression level. **(B–E)** Osteogenic related mRNA (*Alp*, *Opn*, *Ocn*, and *Runx2*) expression level. **(F)** ALP and Alizarin red staining of different surface at day 7 and day 14, respectively. Data are presented as mean ± SD; (*p < 0.05, vs. Ti group).

The ostegenic differentiation of BMSCs on 1Cu-MAO coatings were evaluated by Real time PCR and Western Blot (Figure 8A). The mRNA of *Alp, Opn, Ocn* and *Runx2* were significantly higher in 1Cu-MAO group than the MAO group (*p* < 0.05), the Protein levels of ALP and RUNX2 showed the same trend (Figures 8B–E). The results of ALP and Alizarin red staining showed that Cu-MAO coating can enhance the ALP activity and mineralized nodules formation of BMSCs (Figure 8F).

### 3.3 Evaluation of antibacterial property

**FIGURE 9:**
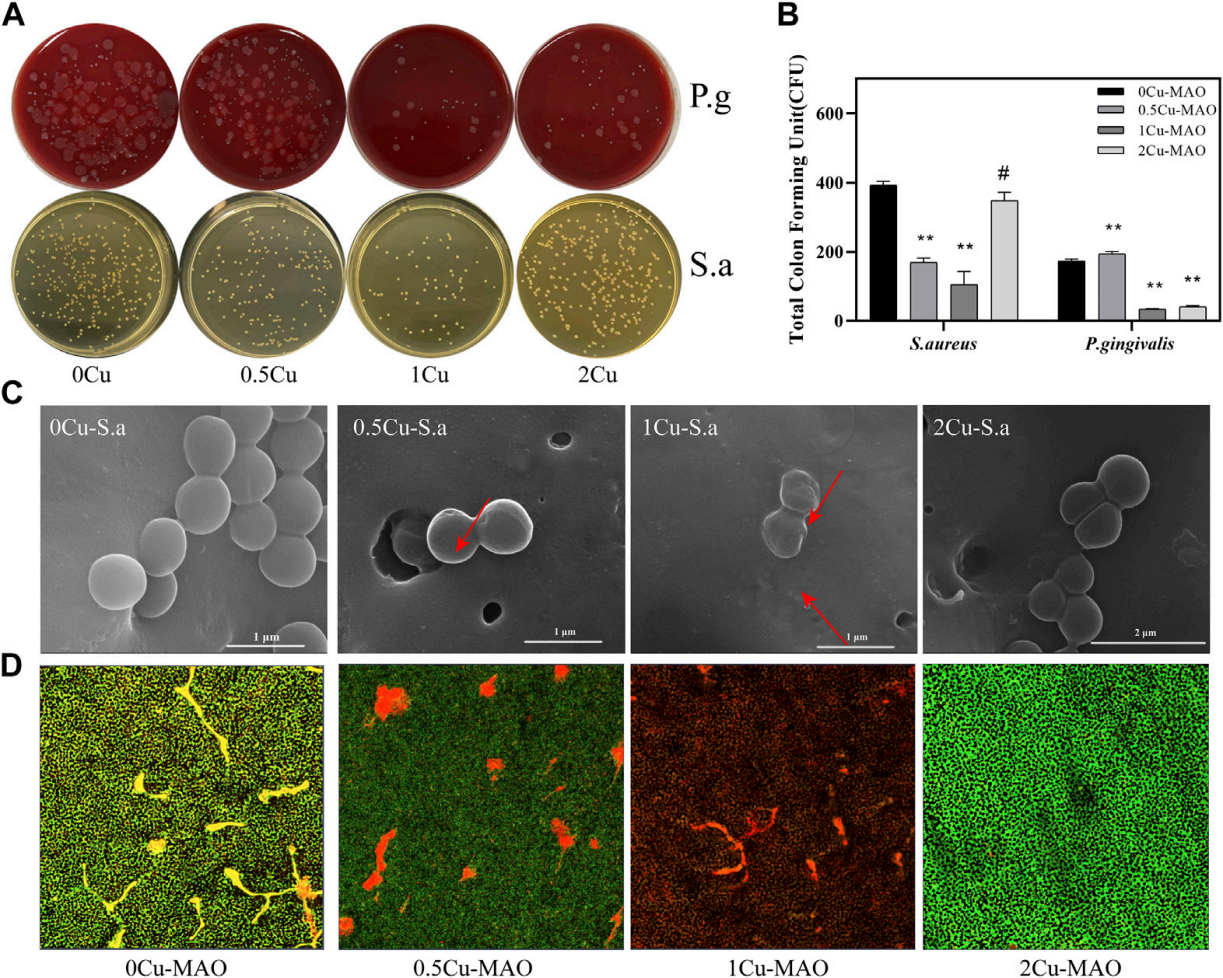
Antibacterial activities of Cu-doped coatings against *S. aureus* and P. gingivalis **(A)**; CFU results after *S. aureus* and *P. gingivalis* were cocultured with the Cu-MAO coating for 24 h. Data are presented as mean ± SD; (**p < 0.01, vs. 0Cu-MAO group); **(B)**; Live/Dead assay **(D)** and the corresponding SEM images **(C)** of *S. aureus* cultured on different surface after 24 h of incubation.

The antibacterial properties of contact-killing of *P. gingivalis* and *S. aureus* for 0Cu-MAO, 0.5Cu-MAO, 1Cu-MAO and 2Cu-MAO after 24 h of incubation were measured as shown in Figure 9. Figures 9A,B shows the total CFU of the MAO coating and Cu-MAO groups. The number of CFU in 1Cu-MAO coating was significantly less than in other coatings (*p < 0.05*). The antibacterial rates of 1Cu-MAO coating against *P. gingivalis* and *S. aureus* were 80.74 and 73.26%, respectively. It indicated that the 1Cu-MAO coating exhibits powerful antibacterial activities, compared to the other coatings.

As showed in Figure 9C, the effects of those Cu-MAO coatings on bacterial morphology and cell wall integrity were observed via SEM. The *S. aureus* grown on the surface of 0Cu-MAO coating are rounded with integrated membranes, while there were some bacterial display collapsed, corrugated and fractured on the surface of Cu-MAO coatings (marked with arrows in Figure 9C). The viability of *S. aureus* cultured on different surfaces were observed by Live/Dead staining, as shown in Figure 9D. The green and red fluorescence intensity reflects the numbers of live and dead bacterial colonies on the coatings, respectively. The biofilm formed on the surface of 0Cu-MAO coating, while viable bacteria stained in green were reduced, and while the dead bacteria stained in red were increased on the Cu-MAO with Cu groups, among which 1Cu-MAO showed the best antibacterial effect in Figure 9D.

## 4 Discussion

MAO is a conventional and effective surface modification process of titanium-based materials based on electrochemical reaction (Kim et al., 2009; Zhang et al., 2020). Under high voltage, porous oxide layer with elements incorporated from the electrolyte solution (Tsutsumi et al., 2021). The content of each element doped in the coating can be controlled by adjusting the electrolyte composition. The micro/nanoscale porous structure and elements doped via MAO onto the surface of the titanium can cnfer the coating bidirectional regulatory functions and improve the implant compatibility and antibacterial property (Hu et al., 2010b).

In this study, Cu-MAO coatings were sucessfully constructed after characterization analyse. Cu-MAO coatings showed the good compatibility and promoted the ostegenic differentiaiton of BMSCs *in vitro* and antibacterial effects on *P. gingivalis* and *S. aureus*, which suggest the potential application in dental implant in future.

Trisodium phosphate was used as the electrolyte to introduce the phosphorus, which can promote the proliferation of osteoblasts (Takebe et al., 2000). Copper acetate was selected as a Cu-containing component, and EDTA was added to chelate with positively charged cupric ions. The addition of KOH can increase the *PH* and conductivity of the solution. Cu-MAO coatings (0.5Cu-MAO, 1Cu-MAO, 2Cu-MAO) were fabricated via adding different mass fractions of copper acetate into electrolytes by MAO (Figures 1, 2). EDS demonstrated the copper has been successfully dopes into TiO_2_ coatings (Table 2; Figure 3A).

In addition, the thickness of Cu-MAO coatings was from 8 to 11 μm (Figure 2). The appropriate dental implant surface topography and chemistry will improve the osseointegration in clinic (Milleret et al., 2019). The surface topography affects the biological functions of peri-implant tissue and bone cells (Elter et al., 2008). Moderately rough surfaces (surface roughness, Sa = 1∼2 μm) (Wennerberg and Albrektsson, 2009) and nanoscale characteristics promote osseointegration (Wang et al., 2021). In our study, the surface topography of 0Cu-MAO, 0.5Cu-MAO and 1Cu-MAO coatings showed the crater like structures, the diameters were less than 10 μm. While 2Cu-MAO coating exhibited larger pore size and lower porosity values (Figure 1D). CLSM results suggested that adding excessive copper could change the morphology and roughness of implants, the surface roughness of the 2Cu-MAO coating dropped from 0.928 to 0.77 μm (Figure 5A), which showed the moderately rough surfaces. Hydrophilic surfaces are beneficial in early phases of osseointegration (Buser et al., 2004), which will be suitable for cells adhension on the implant surface. The contact angles of the Cu-MAO coatings were from 43° to 65.25° (data in brief Figure 5B), which was depend on the copper concentration in coatings. Judging from the results of XPS, the Cu in the 0Cu-MAO, 0.5Cu-MAO and 1Cu-MAO coatings existed mainly as Cu_2_O, while in 2Cu-MAO coating existed primarily in CuO (Figure 4). The cell viability of BMSCs on 0Cu-MAO, 0.5Cu-MAO and 1Cu-MAO coatings was much higher than the Ti group and 2Cu-MAO, which proved that the surface topography of Cu-MAO coatings was relative appropriate and suitable for BMSCs proliferation. Moreover, the osteoblastic markers (ALP, OPN, OCN, and RUNX2) were upregulated in 1Cu-MAO coating after 7 days cultured, which showed the promotion of osseointegration of 1Cu-MAO. All of which showed the good physical and chemical properties and predominant cell biocompatibility and osteogenic ability of 1Cu-MAO in our study. These results illustrated that the addition of excessive copper acetate could evidently modify the surface morphology, roughness, hydrophilia and superficial composition of the coatings. This is due to the addition of copper acetate increased the conductivity of the electrolyte. Compared with 1Cu-MAO, the increase concentration of copper acetate in 2Cu-MAO coating leads the increasing Cu^2+^ concentration of the solution.

$$Cu^{2+}+\mathrm{EDT}A^{4-}↔\mathrm{CuEDT}A^{2-}$$ (3)

According to Eq. 3, copper reacts with EDTA to form CuEDTA^2-^. The increase of copper acetate will increase the CuEDTA^2-^ concentrations. During MAO process, the developed electric field between the anode and the cathode pushes the negatively charged CuEDTA^2−^ complexes toward the anode (Li et al., 2019). At this moment, the main reactions on the anode may be described as follows (Zhang et al., 2018b):

$$\mathrm{Ti}-4e^{-}=Ti^{4+}$$ (4)

$$Ti^{4+}+2O^{2-}=\mathrm{Ti}O_{2}$$ (5)

$$Cu^{2+}+2OH^{-}=\mathrm{Cu}{\left(\mathrm{OH}\right)}_{2}$$ (6)

$$\mathrm{Cu}{\left(\mathrm{OH}\right)}_{2}=\mathrm{CuO}+H_{2}O$$ (7)

According to Eqs 6, 7, the CuO content in the TiO_2_ coating increased with increasing Cu2^+^ concentrations. The ionic release of the 1Cu coatings after immersed in 0.9wt% NaCl for 28 days was examined by ICP mass spectrometry, the result shows no detectable Cu ionic release into the surrounding solution (Figure 6), which suggest the stability and insolubility of the Cu-MAO coating.

Peri-implantitis is an inflammatory disease that promotes the bone absorption after osseointegrate on dental implant, which is the mainly reason that interfere with the long-term stability of the implant. With the high prevalence, peri-implantitis is initial induced by the oral micrograms. Therefore, the antibacterial function of dental implant should be paid major attention in clinic research. *P. gingivalis*, a group of bacteria known as the red complex, is considered to be major pathogen to cause Peri-implantitis (Ito et al., 2014). *S. aureus* is a frequent commensal of the nares and skin and is considered transient oral residents, which is found identified at higher numbers in biofilm obtained from implants with peri‐implantitis than peri‐implant health (Renvert et al., 2014; Zhuang et al., 2016). The biological rationale of *S. aureus*. In peri‐implantitis is the capacity to attach onto titanium surfaces (Harris and Richards, 2004), and contribute to biofilm‐associated on the medical device infections (Costerton et al., 2005). *P. gingivalis* and *S. aureus* were chosen in our study. The OD600, CFU and direct contact results exhibit desirable antibacterial capabilities of Cu-MAO coatings (Figure 9). More interesting, the 1Cu-MAO coating showed the best antibacterial effect among them. The difference of antibacterial efficacy may be mainly ascribed to the effect of chemical configurations of copper in Cu-incorporated TiO_2_ coatings.

As revealed by XPS, the copper in 1Cu-MAO existed mainly as Cu_2_O, while in 2Cu-MAO existed primarily as CuO. It has been widely discussed that Cu^+^ is considered to be a more effective antibacterial agent than Cu^2+^, which is associated with their different pathways of antibacterial activity (van Hengel et al., 2020). The antimicrobial effect of copper oxides (I and II) differs by the oxidation state. For Cu^2+^, its biocidal effect is the involvement of reactive oxygen species. In contrast to Cu^2+^, intracellular proteins have an enhanced affinity toward Cu^+^, which could inactivate the vital enzyme and DNA in bacteria and finally disable their replica capability (Meghana et al., 2015).

## 5 Conclusion

In this work, the influence of copper acetate contents on the microstructure and biological performances of Cu-incorporated TiO2 (Cu-MAO) coatings was systematically studied. *in vitro* The following conclusions could be achieved:

• Different contents of copper element were successfully controlled doped on the surface of porous TiO_2_ coating via facile MAO technique.
• The 1Cu-MAO coating possesses a higher antibacterial capability compared to 0.5Cu-MAO and 2Cu-MAO coatings, which is associated with the Cu_2_O concentration of TiO_2_ coatings.
• The 1Cu-MAO coating exhibits an excellent bioactivity and to be able to promote cell proliferation and adhesion.

## Data Availability

The original contributions presented in the study are included in the article/Supplementary Material, further inquiries can be directed to the corresponding authors.

## References

[B1] Alqattan M. Peters L. Alshammari Y. Yang F. Bolzoni L. J. R. b. (2021). Antibacterial Ti-Mn-Cu alloys for biomedical applications. Regen. Biomater. 8, rbaa050. 10.1093/rb/rbaa050 33732496 PMC7947594

[B2] Brunello G. Elsayed H. Biasetto L. J. M. (2019). Bioactive glass and silicate-based ceramic coatings on metallic implants: Open challenge or outdated topic? Materials 12, 2929. 10.3390/ma12182929 PMC6766230 31510062

[B3] Buser D. Broggini N. Wieland M. Schenk R. Denzer A. Cochran D. (2004). Enhanced bone apposition to a chemically modified SLA titanium surface. J. Dent. Res. 83, 529–533. 10.1177/154405910408300704 15218041

[B4] Costerton J. W. Montanaro L. Arciola C. R. (2005). Biofilm in implant infections: Its production and regulation. Int. J. Artif. Organs 28, 1062–1068. 10.1177/039139880502801103 16353112

[B5] Durdu S. J. C. (2018). Characterization, bioactivity and antibacterial properties of copper-based TiO2 bioceramic coatings fabricated on titanium. Coatings (Basel). 9, 1. 10.3390/coatings9010001

[B6] Elias C. Lima J. Valiev R. Meyers M. J. J. (2008). Biomedical applications of titanium and its alloys. JOM 60, 46–49. 10.1007/s11837-008-0031-1

[B7] Elter C. Heuer W. Demling A. Hannig M. Heidenblut T. Bach F.-W. (2008). Supra-and subgingival biofilm formation on implant abutments with different surface characteristics. Int. J. Oral Maxillofac. Implants 23, 327–334. 18548931

[B8] Gui H. Feng Y. Qiang L. Sun T. Liu L. (2021). Core/shell structural ultra-small gold and amyloid peptide nanocomposites with effective bacterial surface adherence and enhanced antibacterial photothermal ablation. Smart Mat. Med. 2, 46–55. 10.1016/j.smaim.2020.12.001

[B9] Harris L. Richards R. G. (2004). *Staphylococcus aureus* adhesion to different treated titanium surfaces. J. Mater. Sci. Mater. Med. 15, 311–314. 10.1023/b:jmsm.0000021093.84680.bb 15332591

[B10] Heitz-Mayfield L. J. Mombelli A. Implants M. (2014). The therapy of peri-implantitis: A systematic review. Int. J. Oral Maxillofac. Implants 29, 325–345. 10.11607/jomi.2014suppl.g5.3 24660207

[B11] Hu H. Liu X. Ding C. J. J. o. A. (2010). Preparation and *in vitro* evaluation of nanostructured TiO2/TCP composite coating by plasma electrolytic oxidation. J. Alloys Compd. 498, 172–178. 10.1016/j.jallcom.2010.03.147

[B12] Hu H. Liu X. Ding C. J. S. Technology C. (2010). Preparation and cytocompatibility of Si-incorporated nanostructured TiO2 coating. Surf. Coat. Technol. 204, 3265–3271. 10.1016/j.surfcoat.2010.03.028

[B13] Ito T. Yasuda M. Kaneko H. Sasaki H. Kato T. Yajima Y. (2014). Clinical evaluation of salivary periodontal pathogen levels by real‐time polymerase chain reaction in patients before dental implant treatment. Clin. Oral Implants Res. 25, 977–982. 10.1111/clr.12198 23745964 PMC4232322

[B14] Jia B. Mei Y. Cheng L. Zhou J. Zhang L. (2012). Preparation of copper nanoparticles coated cellulose films with antibacterial properties through one-step reduction. ACS Appl. Mat. Interfaces 4, 2897–2902. 10.1021/am3007609 22680307

[B15] Kim D.-Y. Kim M. Kim H.-E. Koh Y.-H. Kim H.-W. Jang J.-H. J. A. b. (2009). Formation of hydroxyapatite within porous TiO2 layer by micro-arc oxidation coupled with electrophoretic deposition. Acta Biomater. 5, 2196–2205. 10.1016/j.actbio.2009.02.021 19299214

[B16] Kim J. Kang I.-G. Cheon K.-H. Lee S. Park S. Kim H.-E. (2021). Stable sol–gel hydroxyapatite coating on zirconia dental implant for improved osseointegration. J. Mat. Sci. Mat. Med. 32, 81. 10.1007/s10856-021-06550-6 PMC8245356 34191141

[B17] Kim S. Park C. Moon B.-S. Kim H.-E. Jang T.-S. (2017). Enhancement of osseointegration by direct coating of rhBMP-2 on target-ion induced plasma sputtering treated SLA surface for dental application. J. Biomater. Appl. 31, 807–818. 10.1177/0885328216679761 27881639

[B18] Li G. Wang Y. Qiao L. Zhao R. Zhang S. Zhang R. (2019). Preparation and formation mechanism of copper incorporated micro-arc oxidation coatings developed on Ti-6Al-4V alloys. Surf. Coat. Technol. 375, 74–85. 10.1016/j.surfcoat.2019.06.096

[B19] Manière C. Lee G. McKittrick J. Chan S. Olevsky E. A. J. A. M. (2020). Modeling zirconia sintering trajectory for obtaining translucent submicronic ceramics for dental implant applications. Acta Mat. 188, 101–107. 10.1016/j.actamat.2020.01.061

[B20] McConoughey S. J. Howlin R. Granger J. F. Manring M. M. Calhoun J. H. Shirtliff M. (2014). Future Microbiol. 9, 987–1007. 10.2217/fmb.14.64 25302955 PMC4407677

[B21] Meghana S. Kabra P. Chakraborty S. Padmavathy N. J. R. (2015). Understanding the pathway of antibacterial activity of copper oxide nanoparticles. RSC Adv. 5, 12293–12299. 10.1039/c4ra12163e

[B22] Mei S. Wang H. Wang W. Tong L. Pan H. Ruan C. (2014). Antibacterial effects and biocompatibility of titanium surfaces with graded silver incorporation in titania nanotubes. Biomaterials 35, 4255–4265. 10.1016/j.biomaterials.2014.02.005 24565524

[B23] Milleret V. Lienemann P. S. Bauer S. Ehrbar M. J. C. I. D. Research R. (2019). Quantitative *in vitro* comparison of the thrombogenicity of commercial dental implants. Clin. Implant Dent. Relat. Res. 21, 8–14. 10.1111/cid.12737 30816636

[B24] Montoya C. Du Y. Gianforcaro A. L. Orrego S. Yang M. Lelkes P. I. (2021). On the road to smart biomaterials for bone research: Definitions, concepts, advances, and outlook. Bone Res. 9, 12. 10.1038/s41413-020-00131-z 33574225 PMC7878740

[B25] Qi S. Wu J. Xu Y. Zhang Y. Wang R. Li K. (2019). Chemical stability and antimicrobial activity of plasma-sprayed cerium oxide-incorporated calcium silicate coating in dental implants. Implant Dent. 28, 564–570. 10.1097/ID.0000000000000937 31517651

[B26] Renvert S. Aghazadeh A. Hallström H. Persson G. R. J. (2014). Factors related to peri-implantitis - a retrospective study. Clin. Oral Implants Res. 25, 522–529. 10.1111/clr.12208 23772670

[B27] Rokosz K. Hryniewicz T. Matýsek D. Raaen S. Valíček J. Dudek Ł. (2016). SEM, EDS and XPS analysis of the coatings obtained on titanium after plasma electrolytic oxidation in electrolytes containing copper nitrate. Materials 9, 318. 10.3390/ma9050318 PMC5503094 28773443

[B28] Sanz M. Chapple I. L. Working Group 4 of the VIII European Workshop on Periodontology (2012). Clinical research on peri-implant diseases: Consensus report of working group 4. J. Clin. Periodontol. 39, 202–206. 10.1111/j.1600-051x.2011.01837.x 22533957

[B29] Smeets R. Henningsen A. Jung O. Heiland M. Hammacher C. Stein J. M. (2014). Definition, etiology, prevention and treatment of peri-implantitis--a review. Head. Face Med. 10, 34. 10.1186/1746-160x-10-34 25185675 PMC4164121

[B30] Su Y. Fu J. Du S. Georgas E. Qin Y.-X. Zheng Y. (2022). Biodegradable Zn–Sr alloys with enhanced mechanical and biocompatibility for biomedical applications. Smart Mat. Med. 3, 117–127. 10.1016/j.smaim.2021.12.004

[B31] Takebe J. Itoh S. Okada J. Ishibashi K. (2000). Biomaterials, Anodic oxidation and hydrothermal treatment of titanium results in a surface that causes increased attachment and altered cytoskeletal morphology of rat bone marrow stromal cells *in vitro* . J. Biomed. Mater Res. 51, 398–407. 10.1002/1097-4636(20000905)51:3<398::aid-jbm14>3.0.co;2-# 10880082

[B32] Thukkaram M. Cools P. Nikiforov A. Rigole P. Coenye T. Van Der Voor P. (2020) Antibacterial activity of a porous silver doped TiO_2 coating on titanium substrates synthesized by plasma electrolytic oxidation, J Appl. Surf. Sci. 500, 144235.1–144235.11. 10.1016/j.apsusc.2019.144235

[B33] Trampuz A. Osmon D. R. Hanssen A. D. Steckelberg J. M. Patel R. J. C. O. Research® R. (2003). Clin. Orthop. Relat. Res. 414, 69–88. 10.1097/01.blo.0000087324.60612.93 12966280

[B34] Tsutsumi H. Tsutsumi Y. Shimabukuro M. Manaka T. Chen P. Ashida M. (2021). Investigation of the long-term antibacterial properties of titanium by two-step micro-arc oxidation treatment. Coatings (Basel). 11, 798. 10.3390/coatings11070798 33361664

[B35] van Hengel I. A. J. Tierolf M. Valerio V. P. M. Minneboo M. Fluit A. C. Fratila-Apachitei L. E. (2020). Self-defending additively manufactured bone implants bearing silver and copper nanoparticles. J. Mat. Chem. B 8, 1589–1602. 10.1039/c9tb02434d 31848564

[B36] Wang F. Li C. Zhang S. Liu H. J. S. Technology C. (2020). Tantalum coated on titanium dioxide nanotubes by plasma spraying enhances cytocompatibility for dental implants. Surf. Coat. Technol. 382, 125161. 10.1016/j.surfcoat.2019.125161

[B37] Wang L.-j. Ni X.-h. Zhang F. Peng Z. Yu F.-x. Zhang L.-b. (2021). Osteoblast response to copper-doped microporous coatings on titanium for improved bone integration. Nanoscale Res. Lett. 16, 146. 10.1186/s11671-021-03602-2 34542720 PMC8452820

[B38] Wang T. Wang J. Wu Y. J. C. S. (2015). The inhibition effect and mechanism of l-cysteine on the corrosion of bronze covered with a CuCl patina. Corros. Sci. 97, 89–99. 10.1016/j.corsci.2015.04.018

[B39] Wei H. Cui J. Lin K. Xie J. Wang X. (2022). Recent advances in smart stimuli-responsive biomaterials for bone therapeutics and regeneration. Bone Res. 10, 17. 10.1038/s41413-021-00180-y 35197462 PMC8866424

[B40] Wennerberg A. Albrektsson T. J. (2009). Effects of titanium surface topography on bone integration: A systematic review. Clin. Oral Implants Res. 20, 172–184. 10.1111/j.1600-0501.2009.01775.x 19663964

[B41] Wu B. Xiong S. Guo Y. Chen Y. Huang P. Yang B. J. M. L. (2019). Tooth-colored bioactive titanium alloy prepared with anodic oxidation method for dental implant application. Mat. Lett. 248, 134–137. 10.1016/j.matlet.2019.04.015

[B42] Zeng J. Wang Y. Sun Z. Chang H. Cao M. Zhao J. (2020). A novel biocompatible PDA/IR820/DAP coating for antibiotic/photodynamic/photothermal triple therapy to inhibit and eliminate *Staphylococcus aureus* biofilm. Chem. Eng. J. 394, 125017. 10.1016/j.cej.2020.125017

[B43] Zhang L. Guo J. Yan T. Han Y. J. A. S. S. (2018). Fibroblast responses and antibacterial activity of Cu and Zn co-doped TiO2 for percutaneous implants. Appl. Surf. Sci. 434, 633–642. 10.1016/j.apsusc.2017.10.169

[B44] Zhang X. Li J. Wang X. Wang Y. Hang R. Huang X. (2018). Effects of copper nanoparticles in porous TiO2 coatings on bacterial resistance and cytocompatibility of osteoblasts and endothelial cells. Mater. Sci. Eng. C 82, 110–120. 10.1016/j.msec.2017.08.061 29025639

[B45] Zhang X. Lu X. Lv Y. Yang L. Zhang E. Dong Z. J. A. A. B. M. (2021). Enhancement of corrosion resistance and biological performances of Cu-incorporated hydroxyapatite/TiO2 coating by adjusting Cu chemical configuration and hydroxyapatite contents. ACS Appl. Bio Mat. 4, 903–917. 10.1021/acsabm.0c01390

[B46] Zhang X. Wu Y. Wang J. Xia X. Lv Y. Cai G. (2020). Microstructure, formation mechanism and antifouling property of multi-layered Cu-incorporated Al2O3 coating fabricated through plasma electrolytic oxidation. Ceram. Int. 46, 2901–2909. 10.1016/j.ceramint.2019.09.284

[B47] Zhu W. Zhang Z. Gu B. Sun J. Zhu L. (2013). Biological activity and antibacterial property of nano-structured TiO2 coating incorporated with Cu prepared by micro-arc oxidation. J. Mat. Sci. Technol. 29, 237–244. 10.1016/j.jmst.2012.12.015

[B48] Zhuang L. F. Watt R. M. Mattheos N. Si M. S. Lai H. C. Lang N. P. J. (2016). Periodontal and peri‐implant microbiota in patients with healthy and inflamed periodontal and peri‐implant tissues. Clin. Oral Implants Res. 27, 13–21. 10.1111/clr.12508 25399962

